# MAVS Positively Regulates Mitochondrial Integrity and Metabolic Fitness in B Cells

**DOI:** 10.4049/immunohorizons.2300038

**Published:** 2023-08-23

**Authors:** Hongsheng Wang, Wenxiang Sun, Javier Traba, Juan Wu, Chen-Feng Qi, Laura Amo, Hemanta K. Kole, Bethany Scott, Komudi Singh, Michael N. Sack, Silvia Bolland

**Affiliations:** * Laboratory of Immunogenetics, National Institute of Allergy and Infectious Diseases, National Institutes of Health, Rockville, MD; † Laboratory of Mitochondrial Biology and Metabolism, National Heart, Lung, and Blood Institute, National Institutes of Health, Bethesda, MD; ‡ Departamento de Biología Molecular, Centro de Biología Molecular Severo Ochoa, Consejo Superior de Investigaciones Científicas–Universidad Autónoma de Madrid, Madrid, Spain; § Department of Nephrology, The People’s Hospital of Zhejiang Province, Hangzhou, China

## Abstract

Activated B cells experience metabolic changes that require mitochondrial remodeling, in a process incompletely defined. In this study, we report that mitochondrial antiviral signaling protein (MAVS) is involved in BCR-initiated cellular proliferation and prolonged survival. MAVS is well known as a mitochondrial-tethered signaling adaptor with a central role in viral RNA-sensing pathways that induce type I IFN. The role of MAVS downstream of BCR stimulation was recognized in absence of IFN, indicative of a path for MAVS activation that is independent of viral infection. Mitochondria of BCR-activated MAVS-deficient mouse B cells exhibited a damaged phenotype including disrupted mitochondrial morphology, excess mitophagy, and the temporal progressive blunting of mitochondrial oxidative capacity with mitochondrial hyperpolarization and cell death. Costimulation of MAVS-deficient B cells with anti-CD40, in addition to BCR stimulation, partially corrected the mitochondrial structural defects and functionality. Our data reveal a (to our knowledge) previously unrecognized role of MAVS in controlling the metabolic fitness of B cells, most noticeable in the absence of costimulatory help.

## Introduction

The ligation of the Ag receptor in B cells initiates a signaling cascade that in certain conditions leads to their proliferation and differentiation into effector cells such as Ab-secreting or memory B cells. Throughout this activation response, mitochondria play a pivotal role in providing biosynthetic and bioenergetic needs. Resting B cells mainly rely on mitochondria to generate ATP for survival (1). Upon stimulation through the BCR, TLR, CD40, or a combination thereof, B cells rapidly reprogram to increase mitochondria-dependent and -independent energy sources including oxidative phosphorylation (OXPHOS) and glycolysis, respectively (2–7). OXPHOS through the TCA cycle is a more efficient process in generating ATP than glycolysis, whereas glycolysis is essential for cells to generate the synthetic metabolites needed for rapid proliferation. Coordinated regulation of these processes over time is thought to be critical for cellular proliferation (8). Akkaya et al. (2) have focused on metabolic changes downstream of B cell activation, describing an immediate increase in both OXPHOS and glycolysis following BCR ligation, although BCR stimulation alone seems to be insufficient to sustain optimal long-term survival (2). Taken together, these data support that the initial mitochondrial reprograming following receptor stimulation precedes changes in gene expression. It remains unclear whether BCR signals might be connected to this mitochondrial reprograming at this early stage.

Other than the conventional view of mitochondria as the cellular “powerhouse,” there is mounting evidence implicating mitochondria as a “signaling hub” for innate immune signals. This has been demonstrated to involve intracellular sensing and signaling of viral nucleic acids through mitochondrial-integrated molecules. Examples of this include signaling from within mitochondria to activate the NLRP3 inflammasome and cGAS-STING and via the cytosolic retinoic acid–inducible gene I (RIG-I) or melanoma differentiation-associated gene 5 (MDA5), which sense viral RNA and activate a mitochondria-localized adaptor, the mitochondrial antiviral signaling protein (MAVS) (9). Activated MAVS monomers undergo self-assembly to form polymers that are described as prion-like filaments in vitro (10) or form MAVS puncta at mitochondria in vivo (11). The MAVS polymers recruit numerous adaptor molecules and kinases leading to expression of type I/III IFNs and proinflammatory cytokines (12–19). Large MAVS polymers were found in systemic lupus erythematosus patients and believed to play a role in production of type I IFNs and pathogenesis of the disease (20). In support of these findings, we recently showed that MAVS is required, in a B cell–intrinsic fashion, to regulate the development of autoimmune germinal centers and the production of autoantibodies in a lupus-prone mouse model (21). Schell et al. (22) also reported that MAVS may regulate spontaneous germinal centers in a genetic background–dependent manner. Yet, the mechanism by which MAVS, an innate antiviral signaling molecule, regulates Ag-dependent B cell activation remains undetermined.

Given that MAVS is known to alter autoreactive B cell activation in vivo (21, 22), we have investigated the requirement for MAVS in various tests of Ag receptor–induced activation in vitro. Studies of mitochondria function in these cells can connect defects in proliferation with the necessity of metabolic fitness. In this study, we report that, following BCR ligation, MAVS-deficient B cells proliferated poorly beyond three divisions, concomitant with detection of mitochondrial dysfunction and damage. Thus, our results support that MAVS plays a positive regulatory role in sustaining mitochondrial quality and in the long-term survival after proliferation initiated by BCR activation.

## Materials and Methods

### Mice

*Mavs*^−/−^ mice were provided by Dr. James Z. Chen at the University of Texas Southwestern Medical Center and later were obtained from The Jackson Laboratory (stock no. 008634). C57BL/6J (B6) mice were purchased from The Jackson Laboratory (stock no. 000664). Chimeric mice were generated by bone marrow reconstitution at a 1:1 ratio of B6 and *Mavs*^−/−^ origins using irradiated (600 rad) *Rag-1*^−/−^ mice (Taconic) as recipients. After 8 wk of reconstitution, mice were euthanized using carbon dioxide in accordance with Animal Research Advisory Committee guidelines (https://oacu.oir.nih.gov/system/files/media/file/2021-06/b5_euthanasia_of_rodents_using_carbon_dioxide.pdf), and B cells were purified for experiments. All mice were maintained under specific pathogen-free conditions. Animal studies were conducted according to an approved protocol by the National Institute of Allergy and Infectious Diseases (NIAID) Animal Care and Use Committee. All methods were approved by the NIAID Animal Care and Use Committee.

### B cell purification

Peripheral lymph nodes including a total of six axillar, cervical, and inguinal lymph nodes and spleen from each mouse were pooled for making single-cell suspensions using slides to smash tissues in 10 ml of HBSS supplemented with 3% FBS. RBCs were lysed with ACK (ammonium-chloride-potassium) lysis buffer (Lonza) followed by filtration with a 70-μm cell strainer. The cells were subsequently incubated with PE-conjugated Abs against CD43 and CD9, followed by purification using an EasySep mouse PE positive selection kit II according to the manufacturer’s instruction (STEMCELL Technologies). The purity of follicular (FO) B cells was ∼95%. All downstream experiments were performed using purified FO B cells.

### CFSE labeling

Purified FO B cells were washed once in PBS, then resuspended at a concentration of 1 × 10^7^/ml in PBS supplemented with 1 μM CFSE (Thermo Fisher Scientific). After incubation for 10 min at room temperature, the cells were washed three times with complete RPMI 1640 medium (Thermo Fisher Scientific, catalog no. 11875093) containing 2 mM l-glutamine, 10% FBS, 50 U/ml penicillin, 50 μM streptomycin, 0.1 mM nonessential amino acids, 1 mM sodium pyruvate, and 50 μM 2-ME.

### B cell stimulation

For proliferation assays, purified B cells labeled with CFSE or without were cultured in complete RPMI 1640 medium for up to 4 d at 37°C under 5% CO_2_. The following reagents were used for stimulation: F(ab′)_2_ goat anti-mouse IgM (10 μg/ml, Jackson ImmunoResearch Laboratories, catalog no. 115-006-020) or anti-CD40 (2 μg/ml, Thermo Fisher Scientific, catalog no. 16-0402-86).

### Flow cytometry

Approximately 1–2 × 10^6^ cells per sample per well were stained with the indicated Abs using U-bottom 96-well plates and with 50 μl per well of Ab dilution for staining and 200 μl per well of buffer (HBSS supplemented with 3% FBS) for washes. Live/dead labeling was performed using eBioscience fixable viability dye eFluor 780 or a SYTOX AADvanced dead cell stain kit (Thermo Fisher Scientific). The cells were analyzed on a LSR II or a LSRFortessa X-20 flow cytometer (BD Biosciences) and by FlowJo (version 10) software (Tree Star).

For the calcium flux assay, B cells were labeled with fluo-3 and Fura Red (Thermo Fisher Scientific), followed by stimulation with anti-IgM F(ab′)_2_ at 10 μg/ml and analysis by flow cytometry.

For intracellular phospho-protein staining, cells were fixed in 4% paraformaldehyde for 20 min at 4°C, followed by washes with PBS and incubation with 90% methanol/PBS for 60 min at 4°C. The cells were washed twice with PBS and stained with the indicated Abs, followed by washes and analyzed by flow cytometry.

For measuring cell proliferation, CFSE-labeled cells were analyzed by flow cytometry. Data were analyzed by FlowJo to calculate the proliferation index (PI), which is the total number of divisions divided by the number of cells that went into division, and division index (DI), which is the average number of cell divisions that a cell in the original population has undergone. Because only responding cells are reflected in the PI, the PI is a more useful value to compare from sample to sample according to FlowJo software.

For measuring mitochondrial membrane potential, ∼2 × 10^5^ cells per well were cultured in 96-well plates in phenol red–free complete RPMI 1640 medium at 37°C for different times. Before harvesting, oligomycin or FCCP was each added to one of the three replicate wells at a final concentration of 6 and 5 μM, respectively, for 10 min at 37°C. An equal volume of medium was added to the third well. The cells were then incubated with 30 nM tetramethylrhodamine methyl ester perchlorate (TMRM) (Thermo Fisher Scientific) and 1 μM SYTOX Blue dead cell stain (Thermo Fisher Scientific) for 30 min at 37°C. The cells were rapidly analyzed by flow cytometry and mean fluorescence intensity (MFI) of TMRM of viable cells was calculated by FlowJo software. The percentage of the maximum mitochondrial membrane potential (MMMP) was calculated using the following equation: 100 × [(MFI_TMRM alone_ − MFI_TMRM+FCCP_)/(MFI_TMRM+oligomycin_ − MFI_TMRM+FCCP_)] (2).

For measuring MitoTracker Green uptake, phenol red–free complete RPMI 1640 medium was used throughout the assay. Stimulated cells were washed and equilibrated with fresh medium at 37°C for 10 min before adding 40 nM MitoTracker Green (Thermo Fisher Scientific) and 1 μM SYTOX Blue dead stain (Thermo Fisher Scientific) for another 30 min. After cells were spun down they were resuspended in 300 μl of fresh medium and immediately analyzed by flow cytometry.

To measure mitochondrial reactive oxygen species (ROS), stimulated B cells were washed and incubated with HBSS with Ca^2+^ and Mg^2+^ supplemented with 1 μM MitoSOX Red at 37°C for 30 min. Cells were washed three times with HBSS with Ca^2+^ and Mg^2+^, followed by incubation with 1 μM SYTOX Blue dead stain at 37°C for 10 min and immediately analyzed by flow cytometry.

### Extracellular flux assay

Cellular oxygen consumption rate (OCR) was measured by using a Seahorse XF96 analyzer as described previously (2). Briefly, stimulated cells (dead cells were eliminated by either low-speed centrifugation or cell sorting) were plated at 5 × 10^5^ per well in 96-well Seahorse plates with Seahorse medium. The following agents were used during the measurement: oligomycin (1 μM), 2,4-dinitrophenol (DNP, 0.1 mM), rotenone (1 μM), antimycin A (1 μM), glucose (10 mM), and 2-DG (50 mM).

### Confocal microscopy and transmission electron microscopy

For confocal microscopy analysis, cells were enriched for viable cells by low-speed centrifugation or cell sorting and then labeled with 150 nM MitoTracker Red CMXRos (Thermo Fisher Scientific) and LIVE/DEAD Green at 37°C for 30 min. The cells were then washed and cytospun onto slides. After fixation and permeabilization, the slides were incubated with rabbit anti-mouse TOM20 Abs (Abcam) and secondary ATTO-647–labeled anti-rabbit Abs (Sigma-Aldrich) and DAPI. Images were collected using a Zeiss LSM880 confocal microscope equipped with a super-resolution Airyscan detector and analyzed by using Huygens and Imaris software.

For transmission electron microscopy analysis, B cells were fixed with Karnovsky fixative solution and processed by the Microscopy Unit at NIAID/National Institutes of Health (NIH) with a Hitachi H-7800 120-kV transmission electron microscope.

### RNA sequencing

Splenic naive FO B cells from B6 and *Mavs*^−/−^ mice were sort purified for B220^+^CD21^lo^CD23^+^ cells and were processed for RNA preparation (termed day 0 cells). In other cases, splenocytes were enriched for FO B cells by depleting CD43^+^ and CD9^+^ cells as described above. The cells were then stimulated with anti-IgM (10 μg/ml) for 24 h. Following sorting for viable CD19^+^ cells, the cells were processed for RNA sequencing (RNA-seq) by the Rocky Mountain Laboratory Genomics Unit of NIAID/NIH. Raw reads were trimmed of adapter sequence using Cutadapt (https://cutadapt.readthedocs.io/en/stable/), and the remaining reads were then filtered for low quality using the FASTX-Toolkit (http://hannonlab.cshl.edu/fastx_toolkit/). Remaining reads were mapped to the GRCm38 genome, using HISAT2. Reads mapping to genes were counted using htseq-count (23). Differential expression analysis was performed using the Bioconductor package DESeq2 (24). Gene set enrichment analysis was performed using Qlucore software and a two-group comparison *t* test.

### Statistical analysis

Student *t* test, one-way ANOVA with a Tukey post hoc test, and the one-sample *t* test with Bonferroni adjustment were used to compare differences between groups of experiments. *p* < 0.05 is thought to be statistically significant.

### Data availability

The RNA-seq data were deposited at the Gene Expression Omnibus (GEO) site with an accession number GSE228413 (https://www.ncbi.nlm.nih.gov/geo/query/acc.cgi).

## Results

### MAVS-deficient B cells show normal early response but impaired proliferation upon BCR stimulation

**FIGURE 1.**
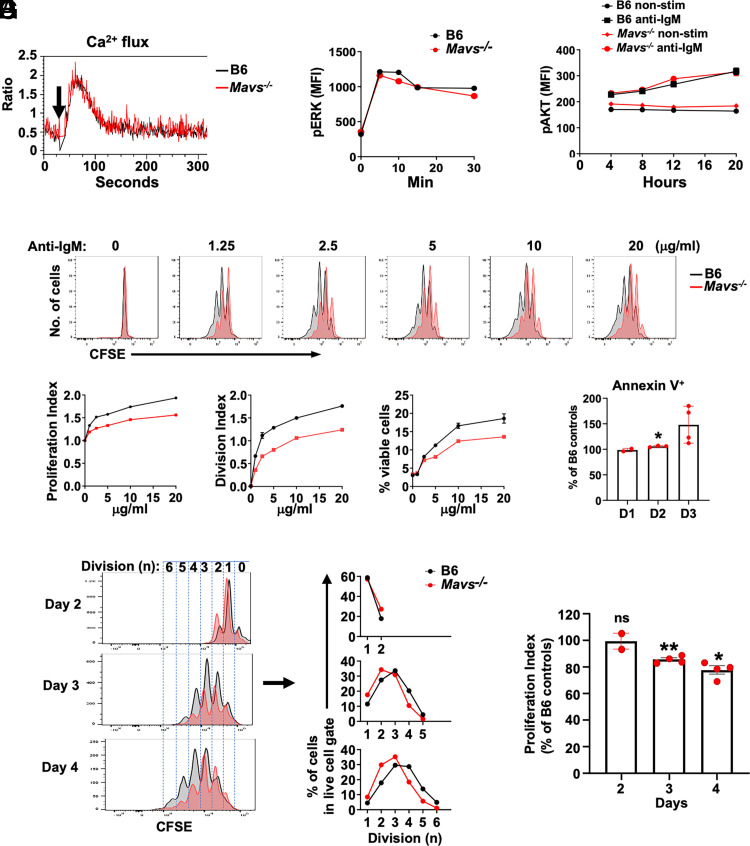
BCR-triggered signaling and proliferation in MAVS-deficient B cells. (**A**–**C**) Purified B cells were stimulated with anti-IgM for the indicated times and measured for calcium influx (A), phosphorylated ERK (B), and phosphorylated AKT (C) by flow cytometry. Data are representative of three independent experiments. (**D**) B cells were labeled with CFSE and stimulated with the indicated concentrations of anti-IgM Abs for 4 d. The viable cells were gated on 7-aminoactinomycin D^−^ cells. (**E**–**G**) CSFE-labeled B cells were stimulated with anti-IgM Abs (10 μg/ml) for the indicated time and analyzed by flow cytometry as in (D). In (B) and (D), line charts are from two mice (means ± SEM) per group. Data are representative of three (A–C) or four independent experiments (D–F). In (E) and (G), each dot represents a mouse. *p < 0.05, **p < 0.01. D1, day 1; D2, day 2; D3, day 3.

To better understand how MAVS deficiency prevents autoimmunity in *Fcgr2b*-deficient mice (21), we focused on B cell activation because previous data implicated a B cell–intrinsic effect. We first analyzed BCR signaling competency in MAVS-deficient FO B cells in vitro. We excluded marginal zone (MZ) B cells (which represent ∼10% of total splenic B cells in a wild-type [WT] mouse) from our B cell preparations throughout this study because of the divergent BCR-mediated cross-linking effects in MZ and FO B cells (25). It was previously shown that MZ B cells undergo apoptosis whereas FO B cells proliferate following BCR ligation (25). Under steady state, it has been shown that the development of FO B cells in *Mavs*^−/−^ mice was normal, even though the MZ B cell compartment was slightly expanded when compared with WT mice (B6 mice throughout this study) (21). We assessed calcium mobilization in response to anti-IgM (F(ab′)_2_) Ab stimulation and found no difference between MAVS-deficient and MAVS-sufficient B cells (Fig. 1A). Furthermore, there was no difference between genotypes in ERK or AKT phosphorylation acutely (Fig. 1B) or up to 20 h (Fig. 1C). These results support that the BCR signaling competency is intact immediately after receptor engagement in the absence of MAVS.

We next analyzed BCR-stimulated proliferation by measuring the dilution of CellTrace CFSE. The proliferation of MAVS-deficient B cells was progressively impaired compared with B6 controls in cultures containing as little as 1.25 μg/ml anti-IgM Abs during a 4-d period (Fig. 1D). The PI and DI increased proportionally with higher amounts of anti-IgM Abs in both MAVS-sufficient and -deficient B cells. However, the PI and DI remained suppressed in MAVS-deficient B cells at all concentrations of stimulant (Fig. 1D), and the number of viable (7-aminoactinomycin D^−^) cells was also lower in MAVS-deficient B cells compared with B6 controls (Fig. 1D). Staining with annexin V, a specific phosphatidylserine-binding protein that has been used to detect apoptotic cells, showed a time-dependent and moderate increase in apoptotic cells in anti-BCR–stimulated MAVS-deficient B cells compared with B6 controls (Fig. 1E). Within the first 2 d of stimulation with anti-IgM Abs, MAVS-deficient and -sufficient B cells appeared to proliferate equally well (Fig. 1F, 1G). However, the proliferation of MAVS-deficient B cells was significantly reduced at days 3 and 4 following stimulation, as represented by the reduced numbers of dividing cells with three or more divisions and an overall reduction of the PI compared with B6 cells (Fig. 1F, 1G). These data collectively suggested that MAVS has a positive influence in survival/proliferation beyond three divisions in Ag receptor–activated B cells.

**FIGURE 2.**
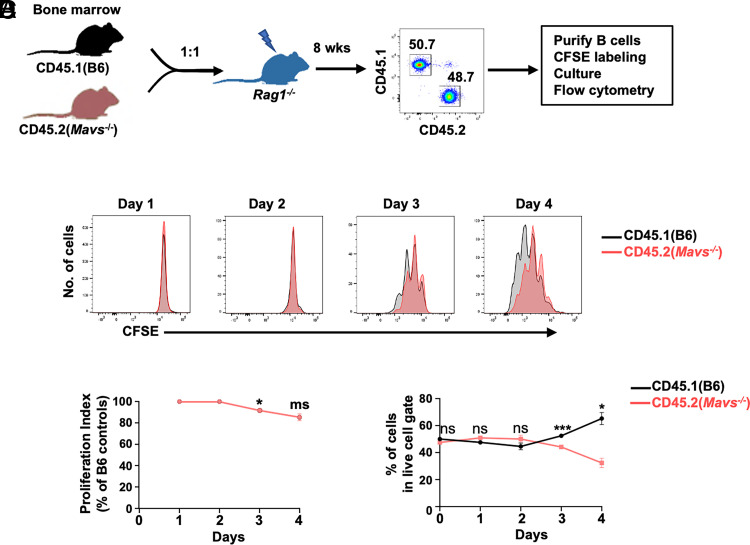
Proliferation of MAVS-deficient B cells in chimera mice. (**A**) Scheme of generation of chimera mice using bone marrow cells of CD45.1 (B6) and CD45.2 (*Mavs*^−/−^) mice at a 1:1 ratio. Peripheral blood cells were analyzed by flow cytometry and were gated on live cells. The numbers on the histogram are percentages of cells falling in each gate. (**B**) Purified B cells from chimera mice were labeled with CFSE and stimulated with anti-IgM (10 μg/ml) for the indicated time, followed by flow cytometry analysis. (**C** and **D**) Summary of two independent experiments as in (B) with multiple mice is depicted. *p < 0.05, ***p < 0.001. ms, marginally significant (*p* = 0.05); ns, not significant.

We further confirmed that the effect of MAVS in B cell proliferation was cell intrinsic by purifying cells from mice reconstituted with mixed bone marrow cells. In these mixed adoptive transfer experiments, MAVS-deficient (CD45.2) and MAVS-sufficient (CD45.1) B cells develop in vivo in the same environment and can be simultaneously activated with anti-IgM Abs in vitro (Fig. 2A). We previously reported the phenotypical analysis of B cells in WT:*Mavs*^−/−^ mixed chimera mice (as shown in supplemental figure 3 in Ref. 21). We observed equal development of WT and *Mavs*^−/−^ B cells in bone marrow, finding similar frequencies in pro-, pre-, and immature B cells. In the periphery, MAVS-deficient mature B cells were overrepresented in the MZ and slightly underrepresented in the FO population compared with MAVS-sufficient B cells. When we tested proliferation of cells purified from these mixed chimeric mice, and consistent with our previous findings, CD45.2^+^ MAVS-deficient B cells showed poorer proliferation beyond three divisions compared with CD45.1^+^ B6 B cells (Fig. 2B, 2C). The frequencies of viable CD45.2^+^ *Mavs*^−/−^ B cells were equivalent to CD45.1^+^ B6 controls in the first 2 d of stimulation but gradually became underrepresented at day 3 and beyond (Fig. 2D). Taken together, these results suggest that the proliferative deficiency of *Mavs*^−/−^ B cells was BCR-dependent and became clear beyond the first 2 d after stimulation.

### MAVS-deficient B cells express perturbed IFN signaling, cell cycling, and metabolic gene programs

**FIGURE 3.**
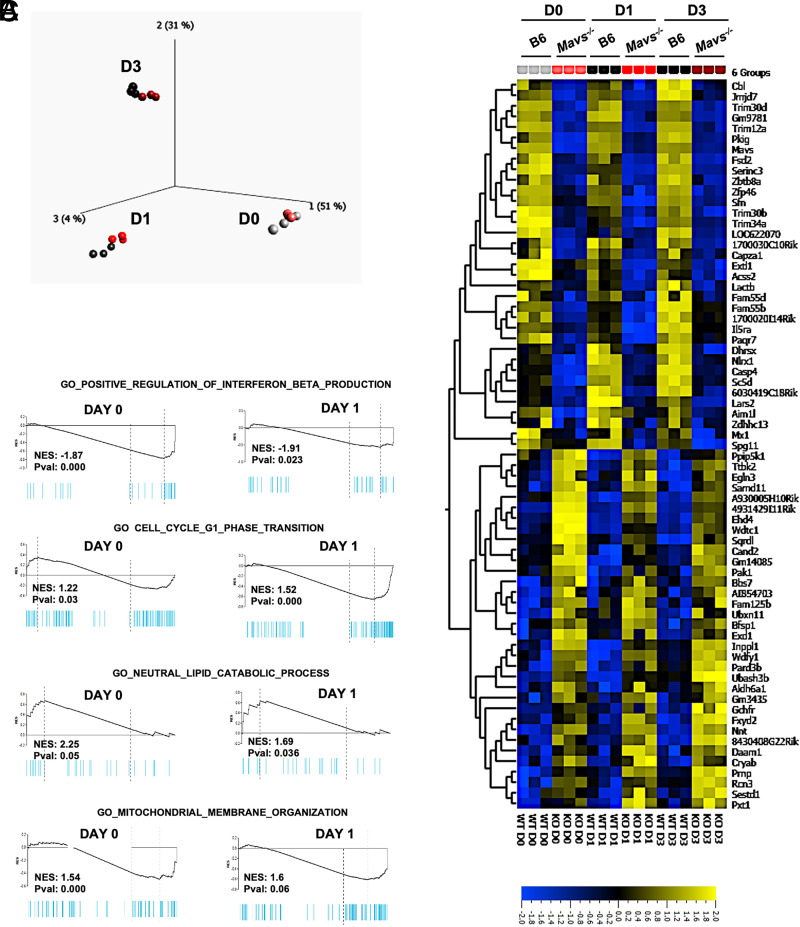
RNA-seq analysis of gene expression profile in B6 and *Mavs*^−/−^ B cells. (**A**) Principal component analysis plot showing variances of the three biological replicates of B6 (gray–dark) and MAVS-deficient (red–dark red) mice that were treated for the indicated time points. (**B**) Heatmap of differentially expressed genes from FO B cells of MAVS-deficient and -sufficient (B6) mice at days 0, 1, and 3. Shown is the scale of ≥2.5-fold for induction or repression. (**C**) Gene set enrichment analysis on differentially expressed genes from day 0 (left panels) and day 1 (right panels) B cells shows enrichment of transcripts involved in regulation of IFN-β production, cell cycle G_1_ phase transition, neutral lipid catabolic process, and mitochondrial membrane organization. D0, day 0; D1, day 1; D3, day 3.

To determine whether MAVS deficiency might result in downstream alterations of gene expression, we performed RNA-seq analysis in resting (day 0) and anti-IgM–stimulated B cells at days 1 and 3. We used principal component analysis to compare expression patterns in MAVS-deficient and -sufficient (B6) B cells collected in all three time points. The data showed distinct patterns that separate the three different conditions (days 0, 1, 3) whereas B6 and MAVS-deficient samples were close to each other within the same stimulation period (Fig. 3A). Consistent with this overall pattern, the numbers of differentially expressed genes (DEGs) between B6 and MAVS-deficient B cells were relatively small (52 genes in day 0, 36 genes in day 1, and 79 genes in day 3) using a cutoff threshold of ≥2.5-fold and a *p* value of ≤0.001. Large subsets of DEGs were common for all three time points (Fig. 3B), an indication that the proliferation defect due to MAVS deficiency, only observed on day 3 and beyond, is not associated with large changes in gene expression. It is noteworthy that none of these DEGs was a recognized NF-κB target gene, an unexpected finding given that MAVS has been shown to activate this transcription factor (19). Gene Ontology analysis revealed top pathways with changes for enriched genes involving IFN-β production, cell cycle, lipid catabolism, and mitochondrial membrane organization (Fig. 3C). Transcriptional changes in IFN, catabolism, and mitochondrial organization genes were consistent both in day 0 and day 1 cells and indicated a persistent deficiency in MAVS-deficient B cells that could not be compensated for by BCR signaling. Changes in cell cycle–connected gene expression were observed in stimulated cells but not in resting B cells. The significantly downregulated IFN-related genes in MAVS-deficient B cells indicated that MAVS may spontaneously stimulate the IFN regulatory factor pathway purportedly to maintain a basal level of IFN signaling. Of note, there was no increase in expression of type I IFN–related genes in anti-BCR–stimulated B6 cells (Supplemental Fig. 1A), which differs from virus-induced MAVS signaling that leads to production of type I IFNs. We further ruled out the possibility that environmental IFN may affect proliferation by coculturing cells with IFNneutralizing Abs (Supplemental Fig. 1B). Thus, the gene expression profile of *Mavs*^−/−^ B cells is consistent with a composite effect of MAVS deficiency on IFN signaling and mitochondria-associated alterations.

### MAVS deficiency induces mitochondrial stress and dysfunction in anti-IgM–stimulated B cells

**FIGURE 4.**
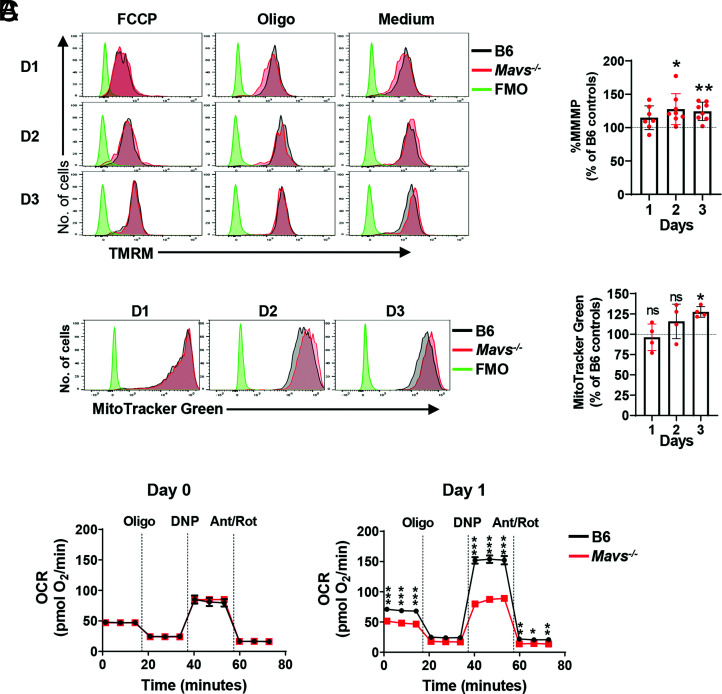
Impaired mitochondrial function in MAVS-deficient B cells. (**A**) Purified B cells were stimulated with anti-IgM (10 μg/ml) for 1–3 d. The cells were then treated with FCCP (5 μM) or oligomycin (6 μM), followed by TMRM (30 nM) and SYTOX Blue dead cell stain. The cells were analyzed by flow cytometry. The left panel is a representative plot gated on viable cells stimulated for 1, 2, and 3 d, and the right panel includes summary data of five independent experiments. Each dot represents a mouse. *p < 0.05, **p < 0.01. (**B**) B cells were stimulated as in (A) and stained with MitoTracker Green. Representative histograms (left panel) and the summary data of two independent experiments (right panel) are shown. Each dot represents a mouse. *p < 0.05. ns, not significant. (**C**) Purified B cells were stimulated with anti-IgM (10 μg/ml) for 1 d or not stimulated (day 0). Viable cells were purified and analyzed by using a Seahorse analyzer to measure oxygen consumption rate (OCR). Representative data of four independent experiments with similar results are shown. Error bars represent triplicate assays. Note that error bars of *Mavs*^−/−^ cells were too small to be seen. *p < 0.05, **p < 0.01, ***p < 0.001. D1, day 1; D2, day 2; D3, day 3; MMMP, maximal mitochondrial membrane potential.

Our gene expression analysis of BCR-activated B cells pointed to alterations in metabolic fitness and mitochondrial membrane organization due to the absence of MAVS, which could be consistent with defects in mitochondria function as described by Akkaya et al. (2). To test this, we carried out a series of mitochondrial functional assays in B cells purified from control and MAVS-deficient mice. The mitochondrial membrane potential, as measured by the mitochondrial dye TMRM, correlates with the capacity of mitochondria to generate ATP by using proton pumps (complexes I, III, and IV). To measure percentages of the MMMP, which is an indicator of cell health and stress (2), we used FCCP, an ionophore uncoupler of OXPHOS, which eliminates mitochondrial membrane potential, and oligomycin, which induces hyperpolarization of mitochondria. *Mavs*^−/−^ B cells showed higher levels of %MMMP than did normal B cells (Fig. 4A), indicating increased mitochondrial stress in MAVS-deficient B cells. MitoTracker Green was previously used to measure the size of mitochondria (26), but a recent study showed that it can also be used to determine mitochondrial stress, particularly in B cells stimulated with anti-IgM Abs (2). Although there was no difference between B6 and *Mavs*^−/−^ B cells in MitoTracker Green uptake in cells stimulated with anti-IgM for 1 d, *Mavs*^−/−^ B cells stimulated with anti-IgM for 2 or 3 d captured more MitoTracker Green than did normal B cells (Fig. 4B). This finding thereby confirmed the elevated mitochondrial stress in *Mavs*^−/−^ B cells at 2 d and beyond following anti-IgM stimulation. Consistent with this result, the basal and maximal mitochondrial respiration, measured as OCR, was significantly decreased 1 d after anti-IgM stimulation in *Mavs*^−/−^ B cells as compared with B6 controls. MAVS deficiency did not have a detrimental effect in OCR in unstimulated cells on day 0, thus confirming the necessity of this adaptor-subsequent cell activation (Fig. 4C). These data collectively suggested that the mitochondria require MAVS to minimize stress and dysfunction following BCR cross-linking.

### Mitochondrial remodeling is impaired in MAVS-deficient B cells

A previous study showed that deficiency of MAVS in mouse embryo fibroblasts resulted in elongated mitochondria (27). To determine whether MAVS could also regulate mitochondrial architecture in B cells, we examined mitochondrial morphology under different conditions.

**FIGURE 5.**
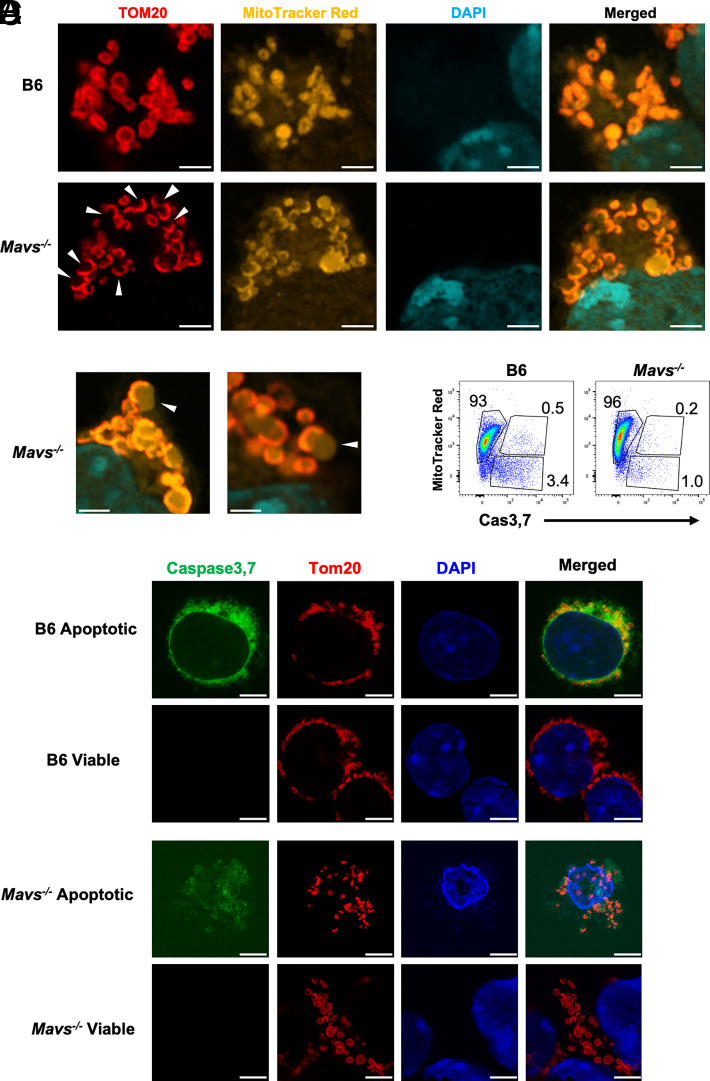
Morphological changes of mitochondria in MAVS-deficient B cells stimulated with anti-IgM. (**A**) Fluorescence imaging analysis showing the mitochondrial morphology and distribution of TOM20 on mitochondria. B cells were stimulated with anti-IgM (10 μg/ml) for 2 d and were labeled with LIVE/DEAD Green (not shown), MitoTracker Red, TOM20, and DAPI. Arrowheads indicate mitochondria with missing TOM20 staining. Scale bars, 2 μm. (**B**) High-power view of *Mavs*^−/−^ B cells with a stretched mitochondrial outer membrane. Scale bars, 1 μm. (**C**) B cells were stimulated with anti-IgM (10 μg/ml) for 2 d and stained with MitoTracker CMXRos Red and caspase-3,7 agents followed by flow cytometry analysis. Numbers are percentages of cells falling in each gate. Data represent three independent experiments. (**D**) Confocal imaging analysis of sorted apoptotic (caspase-3,7^+^) and live (caspase-3,7^−^) cells as in (A). Note that MAVS-deficient apoptotic cells were fragmented during staining on glass slide. Scale bars, 5 μm. Images in (A), (B), and (D) are representative of >14 scanned cells per group. Data in (A), (B), and (D) represent three experiments, and data in (C) represent two independent experiments, all with similar results.

First, B cells were examined by high-resolution Airyscan confocal microscopy with the use of MitoTracker CMXRos Red dye and anti-TOM20 fluorescent Abs. In these images, MitoTracker Red stains the whole mitochondria while detection of TOM20 outlines the mitochondria outer membrane. At 1 d following stimulation with anti-IgM, when the numbers of mitochondria remained low, the shape of mitochondria and the staining pattern of TOM20 were indistinguishable between B6 and *Mavs*^−/−^ B cells (Supplemental Fig. 2A). However, at day 2 following anti-IgM stimulation, when the numbers of mitochondria were significantly increased (Supplemental Fig. 2B), *Mavs*^−/−^ B cells contained abnormal mitochondria with perturbed localization of TOM20 (Fig. 5A). These features included stretching of the outer membrane (more swollen) and polarized (asymmetrical) TOM20 distribution (Fig. 5A, 5B). To exclude apoptosis as an instigator of these changes, we compared apoptotic (caspase-3,7^+^ and MitoTracker Red^−^) with healthy (caspase-3,7^−^ and MitoTracker Red^+^) cells (Fig. 5C) and found that abnormal mitochondria with damaged phenotypes were detected in *Mavs*^−/−^ mice regardless of whether they were alive or apoptotic (Fig. 5D). Therefore, we conclude that MAVS deficiency impaired the mitochondrial integrity in anti-IgM–stimulated B cells before the detection of apoptosis.

**FIGURE 6.**
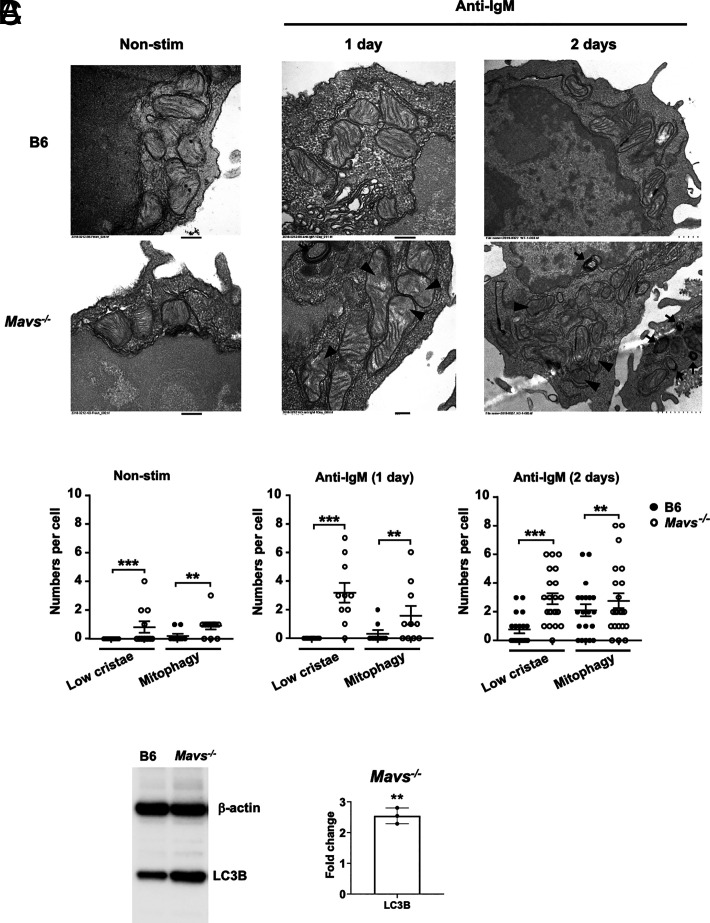
Transmission electron microscopy analysis revealed abnormal mitochondria in MAVS-deficient B cells. (**A**) B cells were either freshly isolated from the spleen (Non-stim) or stimulated with anti-IgM (10 μg/ml) for 1 or 2 d. Arrows indicate mitophagy and arrowheads indicate blurred cristae. Scale bars, 200 nm (left and middle panels) and 1 μm (right panel). (**B**) Quantifications of mitochondria with blurred cristae (low cristae) or mitophagy were made with 10–20 cells from two independent experiments. Each dot represents a cell. **p < 0.01, ***p < 0.001. (**C**) Expression levels of LC3B and β-actin in the indicated cells that were stimulated with anti-IgM Abs for 2 d were analyzed by immunoblotting (left). Quantitative analysis of band intensity is shown in bar chart (right). Each dot represents an independent experiment. Error bars are SD. **p < 0.01.

As a second approach, we examined the mitochondrial structures using transmission electron microscopy. This analysis revealed striking defects of mitochondria in nearly all MAVS-deficient B cells. Compared with B6 controls, MAVS-deficient mitochondria contained distorted cristae in either one pole of the mitochondria or throughout the mitochondrial matrix (Fig. 6A, arrowheads). Mitophagy, defined as an organelle surrounded by multilayers of membrane, was also more common in *Mavs*^−/−^ B cells than in B6 controls (Fig. 6A, arrows). Of note, abnormal mitochondria were found in naive unstimulated *Mavs*^−/−^ B cells, but at much lower frequency than in anti-IgM–stimulated cells (Fig. 6A, 6B). Quantification of mitochondria structural defects, that is, disrupted cristae and mitophagy, in at least 10 cells per condition showed a significant increase of abnormal mitochondria in *Mavs*^−/−^ B cells compared with B6 controls (Fig. 6B). Consistent with the increased mitophagy in MAVS-deficient B cells, the expression levels of LC3B, which correlate with autophagosome formation, were significantly increased in anti-BCR–stimulated *Mavs*^−/−^ B cells compared with controls (Fig. 6C). These data collectively suggested that MAVS-deficient B cells accumulated more damaged mitochondria, which was associated with their compromised functions.

### CD40 signals partially rescue proliferative defects of MAVS-deficient B cells

**FIGURE 7.**
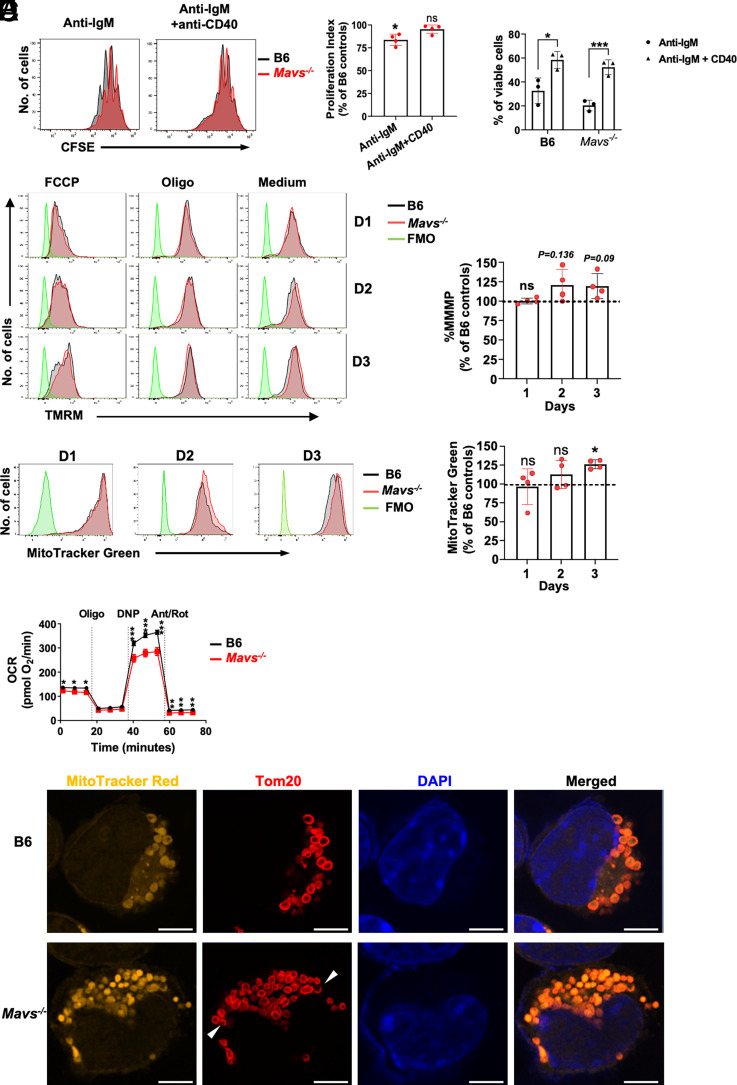
Partial rescue of proliferation in MAVS-deficient B cells by CD40 signals. (**A**) B cells of the indicated mice were stimulated with anti-IgM (10 μg/ml) alone or plus anti-CD40 Abs (2 μg/ml) for 3 d. The right panel is a summary of four independent experiments. *p < 0.05. ns, not significant. (**B**) Viability of stimulated B cells at day 2 was determined by gating on annexin V^−^ cells. *p < 0.05, ***p < 0.001. (**C** and **D**) B cells of B6 and *Mavs*^−/−^ mice were stimulated with anti-IgM plus anti-CD40 as in (A) for 1–3 d. %MMMP and MitoTracker Green staining were performed as in Fig. 4A and 4B, using the same FO B samples and analyzed simultaneously. The left panel is a representative FACS profile gated on viable cells done in parallel with Fig. 4A and 4B (left panels). The right panel shows summary data of four mice from three independent experiments (*p < 0.05. ns, not significant). (**E**) Purified B cells were stimulated with anti-IgM (10 μg/ml) plus anti-CD40 (2 μg/ml) for 1 d. Viable cells were purified and analyzed by using a Seahorse analyzer to measure OCR. Representative data of two independent experiments with similar results are shown. Error bars represent triplicate assays. *p < 0.05, **p < 0.01, ***p < 0.001. (**F**) Confocal imaging analysis of B cells stimulated with anti-IgM plus anti-CD40 for 2 d. The cells were labeled with LIVE/DEAD Green (not shown), MitoTracker Red, TOM20, and DAPI. Scale bars, 5 μm. Data are representative of two independent experiments. Images are representative of >14 scanned cells per group. D1, day 1; D2, day 2; D3, day 3.

CD40 is an important costimulatory molecule for B cell activation and differentiation. Deficiency of CD40 significantly impairs the development of germinal centers and the generation of high-affinity Abs (28). CD40 signals have previously been shown to be efficient in rescuing anti-IgM–induced mitochondrial dysfunction (2). We found that costimulation of B cells with anti-IgM plus anti-CD40 Abs increased the PI in *Mavs*^−/−^ B cells compared with cells stimulated with anti-IgM alone (Fig. 7A). The viability of MAVS-deficient B cells was also significantly increased following anti-IgM plus anti-CD40 stimulation compared with anti-IgM alone (Fig. 7B). Moreover, costimulation with CD40 reduced the gap in the levels of %MMMP and MitoTracker Green uptake, which were not significantly different at day 1 and 2, although they were still significantly higher in *Mavs*^−/−^ compared with B6 controls at day 3 (Fig. 7C, 7D, Supplemental Fig. 3). Thus, MAVS-deficient B cells were relieved from mitochondrial stress by CD40 engagement just as was observed with WT cells. However, this second signal relief was not sufficient to restore complete mitochondrial fitness in MAVS-deficient cells, as they showed some stress at late time points compared with controls. Mitochondrial OCR remained lower in *Mavs*^−/−^ B cells compared with B6 controls under conditions that include costimulation, but again differences were less pronounced than those observed with anti-IgM alone (compare Fig. 7E with Fig. 4C, right panel). Morphological analysis with high-resolution confocal microscopy showed that mitochondria of MAVS-deficient cells had some polarization of TOM20 distribution when costimulated with anti-CD40 (Fig. 7F), but the frequency was markedly lower compared with what was observed with anti-IgM alone (Fig. 5A, 5B). These data collectively suggested that a second signal from CD40 could increase the quality of mitochondria and compensate for part of the proliferative defect caused by MAVS deficiency.

Akkaya et al. (2) reported that anti-IgM stimulation caused elevation in mitochondrial ROS levels that could be measured by the uptake of the reagent MitoSOX. They demonstrated that an additional stimulant such as CpG prevented this type of mitochondrial stress. The presence of cognate T cells reduced ROS levels, presumably because of CD40 engagement in B cells. Thus, we tested the effect of MAVS in mitochondrial ROS levels by measuring MitoSOX uptake in B6 and *Mavs*^−/−^ cells and in various conditions. Regardless of genotype, we observed markedly increased levels of MitoSOX in anti-IgM–treated B cells but not in the presence of anti-CD40 (Supplemental Fig. 4A, 4B). We observed no major differences between B6 and *Mavs*^−/−^ cells in the MitoSOX assay while confirming diminished proliferation in *Mavs*^−/−^ cells from the same experiment (Supplemental Fig. 4C). This result suggests that, in absence of MAVS, mitochondria have functional deficiencies that go beyond elevated cellular oxidative stress.

## Discussion

In this study, we uncover a (to our knowledge) previously unrecognized role of MAVS in regulating mitochondrial integrity and function in B cells. MAVS-deficient B cells proliferated poorly beyond three divisions following BCR ligation. This was associated with reduced mitochondrial functions including decreased OXPHOS and increased mitochondrial %MMMP and evidence of mitochondrial stress. MAVS-deficient mitochondria exhibited a damaged phenotype including stretching of the outer membrane, polarized TOM20 expression, disorganized cristae, and mitophagy. As a result, expression levels of genes involving IFN signaling, catabolism and mitochondrial organization, and cell cycling were altered in MAVS-deficient B cells. These data identify a regulatory role for MAVS to sustain mitochondrial integrity and metabolic fitness in B cells.

It is well demonstrated that MAVS activation by viral RNA leads to production of IFNs and proinflammatory cytokines (12–19). Differing from this dogma, there was no increase in expression of type I IFN–related genes in anti-BCR–stimulated B cells (Supplemental Fig. 1). In contrast, our data suggested the existence of a previously unrecognized BCR/MAVS/mitochondria axis purportedly to fuel B cell proliferation. Freshly isolated MAVS-deficient B cells did not differ from MAVS-sufficient B cells in mitochondrial respiration measured by OCR. However, we detected a reduction of OCR in MAVS-deficient B cells at 1 d following BCR stimulation. We did not observe a difference in proliferation between normal and MAVS-deficient B cells until day 3 following anti-BCR stimulation. This could be due to a lower energy demand in the early phase of proliferation because B cells stimulated with anti-BCR for 3 d clearly exhibited more oxygen consumption than did the cells stimulated for 1 d (Supplemental Fig. 2C). However, this pattern of oxygen consumption quickly changed in the exponential growth phase when MAVS-deficient B cells showed evidence of more mitochondrial stress, which severely impaired the third and fourth divisions and beyond. This explanation is consistent with the observation that the elevated levels of mitochondrial %MMMP and MitoTracker Green uptake were more clear in MAVS-deficient B cells at day 2 following anti-IgM stimulation. The detection of damaged mitochondria that were expanded from naive (day 0) to day 1 and day 2 after anti-BCR stimulation was consistent with these measurements. Collectively, these results suggest that the defective proliferation of MAVS-deficient B cells was likely a result of mitochondrial dysfunction and metabolic unfitness.

Previous studies suggested that a second signal, such as provided by CD40, in addition to the first signal, the BCR signal, is essential for BCR-triggered optimal proliferation and differentiation (2, 29). Stimulation of naive B cells with CD40L plus IL-4 induced expression of genes involved in metabolic reprogramming and mitochondrial remodeling, which results in a rapid doubling of mitochondrial numbers within 24 h (6). While the changes of gene expression programs in B cells stimulated with anti-IgM plus anti-CD40 were not investigated in this study, triggering both BCR and CD40 simultaneously is expected to strongly stimulate the NF-κB pathway. NF-κB activation is known to induce expression of hundreds of nuclear-encoded mitochondrial genes (30) and many of them could protect mitochondria from damage in suboptimal conditions. This protective effect could explain the fact that mitochondrial defects associated with MAVS-deficiency were partially corrected by CD40 signaling at both morphological and functional levels (Fig. 7). However, NF-κB activation doesn’t seem to explain the functional gap between MAVS-deficient and MAVS-sufficient B cells, as we don’t see differential expression of NF-κB-target genes between these cells either in stimulated or resting conditions (Fig. 3).

In the context of B cell activation in vivo, such as in germinal centers, if a second signal is available, it could bypass the need for MAVS to maintain mitochondria integrity, thus allowing MAVS-deficient B cells to differentiate into Ab-secreting plasma cells. However, when second signals are scarce and weak, such as in the early stages of an autoimmune disease, MAVS deficiency could significantly impact B cell activation and renders “protection” to disease development, as observed in MAVS and FcγR2b double deficient mice (21). Therefore, the BCR-MAVS-mitochondria signaling axis functions as a primary pathway with potential to synergize with second signals to drive B cell proliferation and differentiation optimally.

Previous studies showed that overexpression of MAVS in HeLa cells induced mitochondrial fragmentation (31) whereas deficiency of MAVS in mouse embryo fibroblasts resulted in elongated mitochondria (27). Stimulation of fibroblasts with poly(I:C) to mimic dsRNA induced formation of shorter mitochondria, thereby reinforcing that MAVS activation may modulate mitochondrial dynamics (32). How MAVS regulates mitochondrial morphology and remodeling remains elusive but may involve the interaction of MAVS with other mitochondrial remodeling proteins. Evidence suggests that MAVS interacts with various other molecules that regulate outer mitochondria membrane fusion (15, 18, 33). Deletion of TOM70, one of these factors, in cardiomyocytes induces abnormal mitochondria characterized by blurred cristae, swelling, and fragmentation (34), which is a phenotype remarkably resembling MAVS-deficient mitochondria in B cells (Figs. 5, 6). We speculate that MAVS may participate in a yet unknown process to reorganize mitochondrial suborganelle domains required to rapidly boost energy production in B cells. More studies are warranted to evaluate this hypothesis.

In summary, our research suggests that MAVS deficiency causes proliferative impairment in the exponential growth phase of anti-BCR–stimulated B cells concomitant with mitochondrial dysfunction and damage. Interestingly, BCR cross-linking did not enhance type I IFN expression, a phenomenon clearly different from virus-induced MAVS signaling. Although signals second to the BCR such as CD40 could partially restore the mitochondrial function and rescue proliferation in MAVS-deficient B cells, a condition where second signals are scarce would be expected to impact B cell activities in the absence of MAVS. This study supports the role of MAVS in B cell activation with implications in autoimmunity. Future studies are warranted to define the molecular mechanisms of how BCR signaling regulates MAVS to control mitochondrial functions.

## Supplementary Material

Supplemental Figures 1 (PDF)
