# Supplementary material for: MAVS Positively Regulates Mitochondrial Integrity and Metabolic Fitness in B Cells
Source: Immunohorizons. 2023 Aug 23;7(8):587–99. doi: 10.4049/immunohorizons.2300038 (PMC10587501; doi:10.4049/immunohorizons.2300038)
Supplement: Supplemental Figures 1 (PDF) [file IH_2300038_Supplemental_1.pdf]

A

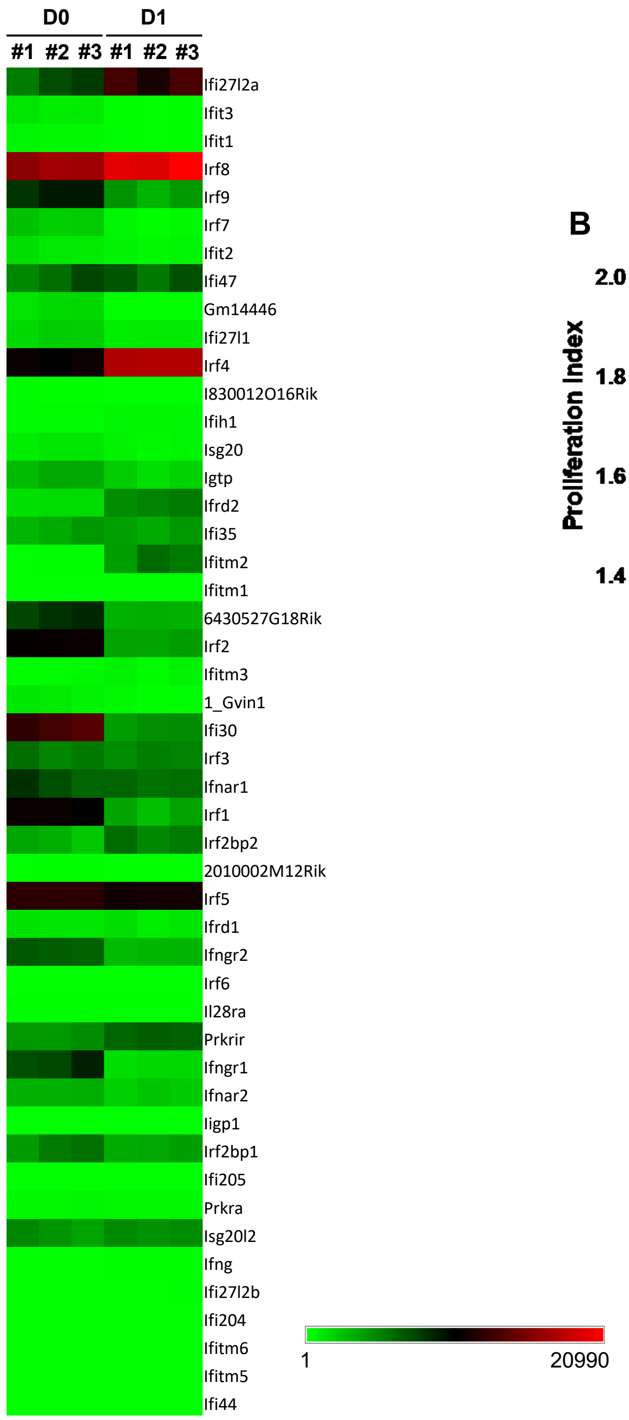

B

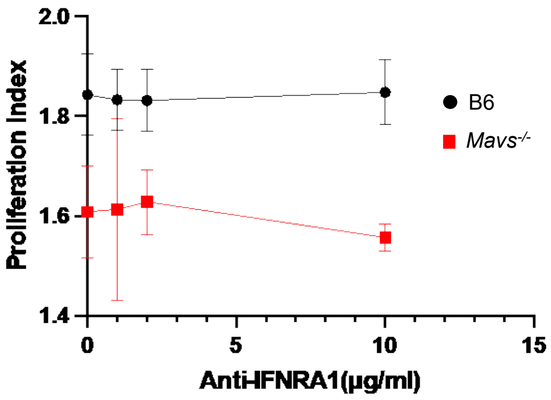

Figure S1. IFN is not involved in anti-IgM stimulated B cells. (A) Expression of IFN-related genes in B cells of B6 mice stimulated with anti-IgM (10 µg/ml) for 1 day (D1) compared with non-stimulated B cells (D0). RNA-seq analysis was done as in Fig. 3. (B) Blocking IFNR did not alter proliferation of MAVS-deficient B cells. CFSE-labeled B cells of the indicated mice were stimulated with anti-IgM (10 µg/ml) in the presence of 1, 2 or 10 µg/ml of anti-IFNR1 neutralizing antibodies for 4 days. Cells were analyzed by flow cytometry. Graph includes data from three separate experiments with six samples per condition and genotype. Two-way ANOVA analysis shows genotype factor $p<0.001$, antibody blocking factor $p=0.88$.

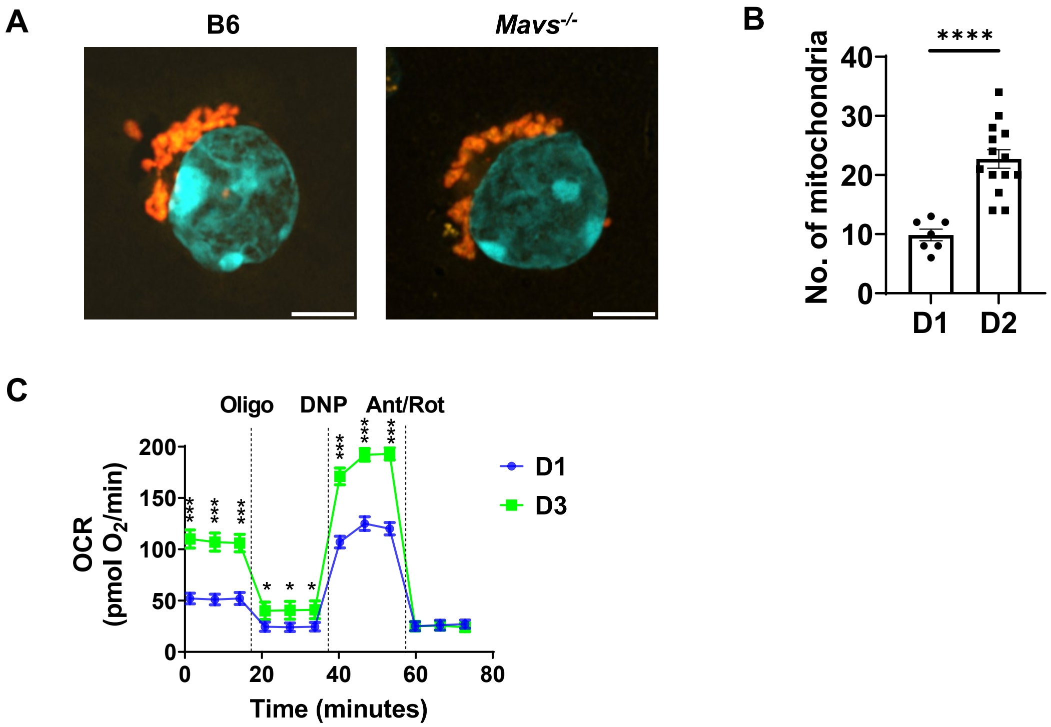

Figure S2. Changes in mitochondria and oxygen consumption in anti-IgM-stimulated B cells. (A) The mitochondrial damage in MAVS-deficient B cells was not profound at 1 day after anti-IgM stimulation. B cells of B6 and *Mavs*$^{-/-}$ mice were stimulated for 1 day with anti-IgM antibodies (10 μg/ml), followed by staining with MitoTracker Red (yellow), anti-TOM20 (red) and DAPI (blue). A Live-Dead cell dye (Green) was used to exclude dead cells from analysis. Scale bars, 5 μm. Images are representative of more than seven scanned cells per group. Data are representative of two independent experiments. (B) Mitochondrial numbers increase following anti-IgM stimulation. Purified B cells from B6 mice were stimulated with anti-IgM antibodies (10 μg/ml) for 1 (D1) or 2 (D2) days. As in (A), the cells were stained with MitoTracker Red, anti-TOM20 and DAPI and analyzed by confocal microscopy. The numbers of mitochondria were counted using serial images from individual cells. Each dot represents a cell. ****p<0.0001. (C) Oxygen consumption increases in B cells stimulated with anti-IgM antibodies. Purified B cells from B6 mice were stimulated with anti-IgM antibodies (10 μg/ml) for 1 (D1) or 3 (D3) days. OCR was measured by a Seahorse analyzer. Error bars are triplicate assays. Data represents two independent experiments with similar results. ***p<0.001, **p<0.01, *p<0.05.

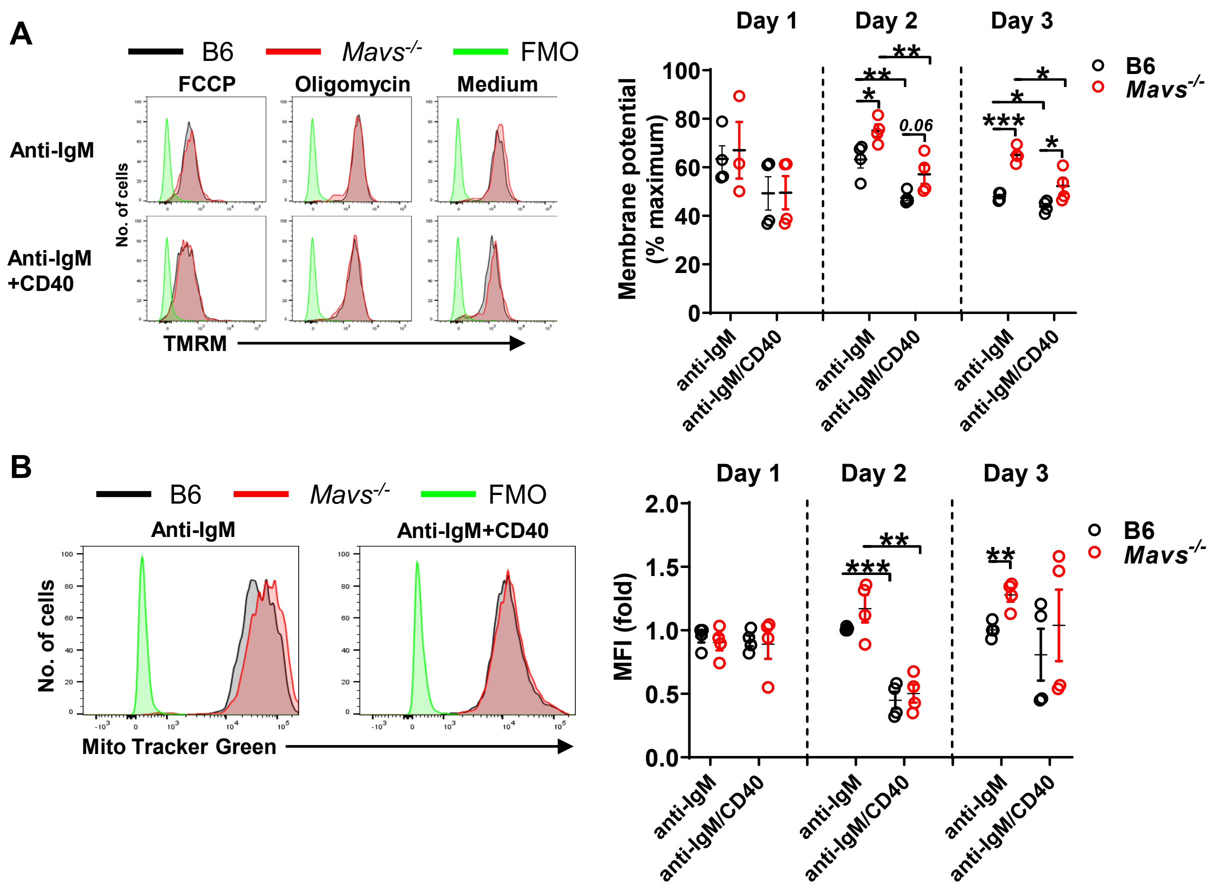

Figure S3. Impaired mitochondrial function of MAVS-deficient B cells is partially rescued by a CD40 signal. Purified B cells were stimulated with anti-IgM (10 μg/ml) or anti-IgM (10 μg/ml) plus anti-CD40 (2 μg/ml) for 1 to 3 days as in Figure 7C and D. The cells were then treated with FCCP (5 μM) or oligomycin (6 μM), followed by TMRM (30 nM) and Sytox Blue dead Cell Stain. The cells were analyzed by flow cytometry. The left panel is a representative plot gated on viable cells stimulated for 2 days (similar to Fig. 4A) and the right panel is the summary data of two independent experiments with four mice. Each dot represents a mouse. (B) B cells were stimulated as in (A) and stained with MitoTracker Green. Histograms in the left panel are representative samples of two-day treatment (similar to Fig. 4B) and the summary data of two independent experiments with four mice are shown in the right panel. Each dot represents a mouse. *p<0.05, **p<0.01, ***p<0.001.

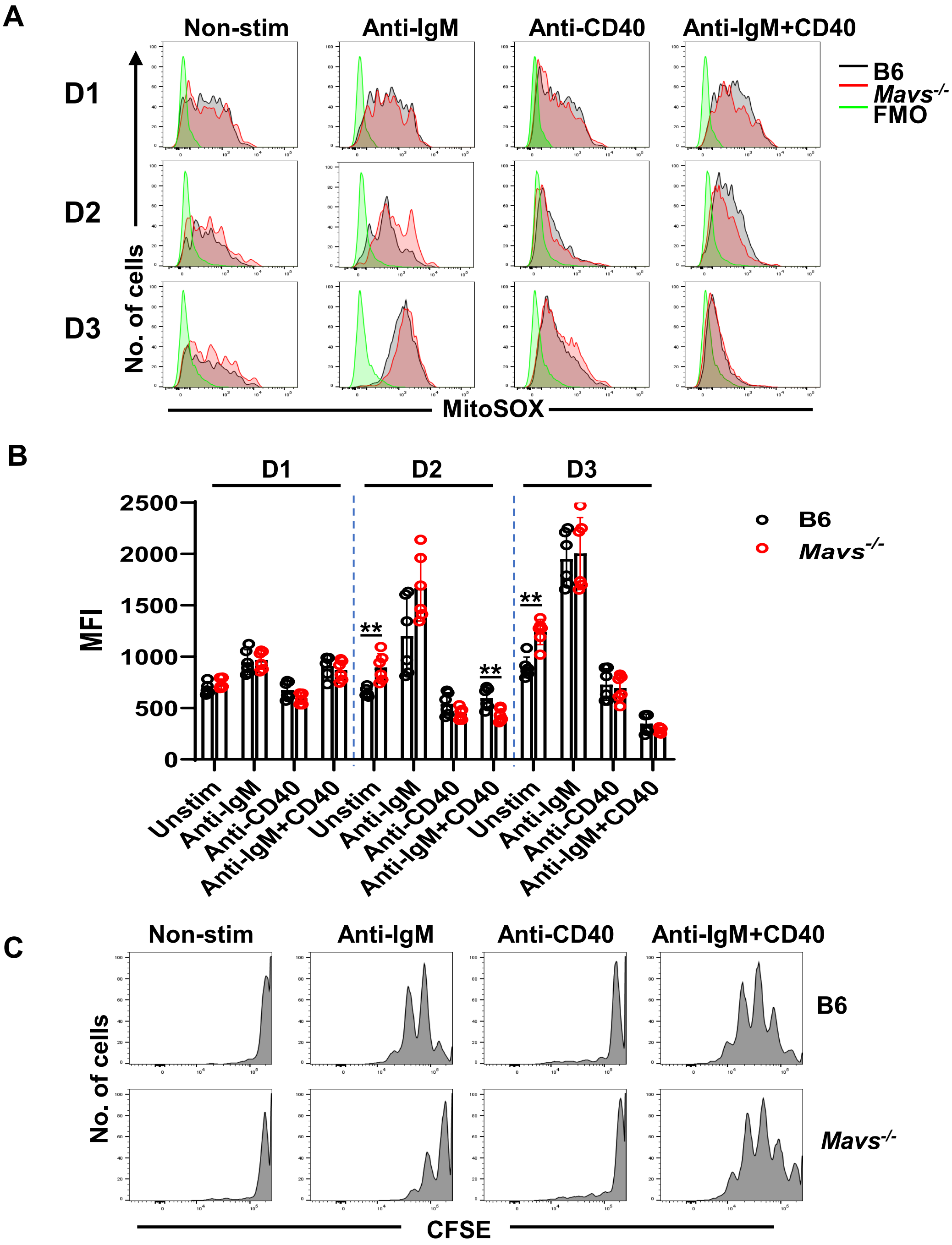

Figure S4. MitoSOX detection of mitochondrial reactive oxygen species (ROS) production in B6 and *Mavs*$^{-/-}$ B cells. Purified B cells were stimulated with anti-IgM (10 μg/ml), anti-CD40 (2 μg/ml) or anti-IgM plus anti-CD40 for 1-3 (D1-D3) days. The cells were then labeled with MitoSOX and SYTOX Blue Dead Stain for 30 and 10 min, respectively, and were analyzed by flow cytometry. (A) Representative histograms showed stimulation and time of different cells. (B) Mean fluorescence intensity (MFI) of MitoSOX in different samples. Symbols represent individual samples from two separate mice per group in each of two independent experiments. $^{**}p<0.01$. (C) Proliferation of the same cells as in (A) was analyzed at day 3 by flow cytometry.
